# Dysferlin Protein–Protein Interaction Pathways in the Organ of Corti and Spiral Ganglion Intersect with Alzheimer’s Protein Pathways

**DOI:** 10.3390/ijms26199559

**Published:** 2025-09-30

**Authors:** Marian J. Drescher, Dennis G. Drescher, Khalid M. Khan, James S. Hatfield, Darshi Hemani

**Affiliations:** 1 Laboratory of Bio-Otology, Department of Otolaryngology, Wayne State University School of Medicine, Detroit, MI 48201, USA; 2 Department of Biochemistry, Microbiology and Immunology, Wayne State University School of Medicine, Detroit, MI 48201, USA

**Keywords:** dysferlin protein–protein interactions, surface plasmon resonance, tectorial membrane, FKBP8, BCL2, ryanodine receptors, Alzheimer’s disease

## Abstract

Dysferlin direct protein–protein interactions (PPI) previously have been elucidated with surface plasmon resonance (SPR) and predicted to underlie membrane repair in mechanotransducing myofibrils. In mechanotransducing inner ear hair cells, dysferlin is detected with Z-stack confocal immunofluorescence in the stereocilia and their inserts in the tectorial membrane (TM) co-localizing with FKBP8, consistent with the SPR determination of tight, positively Ca^2+^-dependent interaction. FKBP8, a direct binding partner of mechanotransducing TMC1, when overexpressed, evokes an elevation in anti-apoptotic BCL2, inhibition of ryanodine receptor (RYR) activity, and a consequent reduction in Ca^2+^ release. RYR3 has now been immunolocalized to the tip of the TM in close association with a third-row outer hair cell (OHC) stereociliary BCL2-positive insertion. Dysferlin, annexin A2, and Alzheimer’s proteins BACE1 and amyloid precursor protein (APP) are also accumulated in these stereociliary insertions. RYR2 and RYR1 have been immunolocalized to the TM core, in position to influence TM Ca^2+^. Dysferlin PPI pathways also intersect with AD protein pathways in the spiral ganglion (SG). Dysferlin segregates with FKBP8, BACE1, and RYR3 in the interiors of SG type I cell bodies. RYR1, RYR2, PSEN1, BCL2, and caspase 3 are primarily confined to plasma membrane sites. RYR3 pathways traverse the plasma membrane to the cell body interior. Western analysis of dysferlinopathy proteins links FKBP8 and BCL2 overexpression with RYR inhibition, indicative of dysferlin targets that are ameliorative in AD.

## 1. Introduction

Two potentially related major issues at the auditory periphery, which are incompletely clarified, are (1) the major source of Ca^2+^ utilized in hair cell mechanotransduction and (2) molecular targeting in the organ of Corti (OC) and spiral ganglion (SG) in Alzheimer’s disease (AD). We suggest that a dysferlin protein–protein interaction (PPI) pathway, as recently described for mechanosensory myofibrils [1], may inform both issues for the mechanosensory mammalian cochlea of the adult. Dysferlin is recognized as a membrane-targeting protein required for membrane repair [1]. We have demonstrated with surface plasmon resonance (SPR) that the carboxy terminus of dysferlin directly interacts with the carboxy terminus of anti-apoptotic protein FKBP8, a tight, positively Ca^2+^-dependent coupling [2], as predicted by the binding of FKBP8 to otoferlin [3], another member of the ferlin protein family. Further, the carboxy terminus of FKBP8 is recognized as a direct binding partner of the carboxy terminus of TMC1, a hair cell mechanotransduction channel component in the cochlea [4]. The dysferlin carboxy terminus would compete with the TMC1 carboxy terminus for binding to the carboxy terminus of FKBP8, consistent with the high-fidelity co-localization of dysferlin with TMC1 observed in myofibrils [2]. FKBP8 has previously been detected in the cochlear tectorial membrane (TM) at P6 in rodents [5]. FKBP8 directly couples to BCL2 [6,7], which in turn directly interacts with the ryanodine receptor(s) (RYR) [8], a potential source of cellular Ca^2+^ in mechanosensory systems. Within the OC, the tectorial membrane has been considered a possible source of Ca^2+^ required for hair cell mechanotransduction [9], without the identification of a physiological mechanism, particularly given that the tectorial membrane heretofore has been considered to be cellularly inert [10].

The relationship between hearing impairment and Alzheimer’s disease (AD) is recognized as highly relevant [11,12] but still poorly understood [13], but it may very well include ryanodine receptor targeting [14]. In order to define molecular deficits underlying early-onset hearing loss in the cochlea contributing to AD [11], a prerequisite first step is the characterization and localization of Alzheimer’s disease-related proteins in the wildtype whose mutation occurs in AD. Although the expression of these proteins in the cochlea has not been studied in detail, an exception is the investigation of BACE1, β-secretase 1, the enzyme responsible for the cleavage of amyloid [β] precursor protein (APP) in AD, determined to be required for hearing in wildtype compared to BACE1-/- mice [15]. A positive immunosignal for BACE1 was observed by Dierich et al. [15] in both the boutons of efferent olivocochlear fibers and post-synaptic afferent SG fibers, suggesting neural sites of action.

In the present investigation, sites of dysferlin (PPI) pathways were examined with immunofluorescence Z-stack confocal microscopy in the cochleae of adult wildtype rats and compared with the localization of wildtype Alzheimer’s disease (AD) proteins BACE1, TAU, PSEN1, caspase 3, and APP. Evidence has been obtained that wildtype proteins for AD are expressed in hair cell stereocilia, their inserts into the tectorial membrane, and SG neurons. A possible physiological mechanism for Ca^2+^ regulation by the tectorial membrane would be suggested by the interaction of hair cell stereocilia inserts with TM ryanodine receptors. Preliminary reports have appeared [16,17,18,19].

## 2. Results

### 2.1. Hair Cell Proteins in Stereocilia Embedded in the Tectorial Membrane: Dysferlin, FKBP8, BCL2, RYRs, and Annexin A2 Form a Molecular Pathway to Regulate Ca^2+^

**Figure 1:**
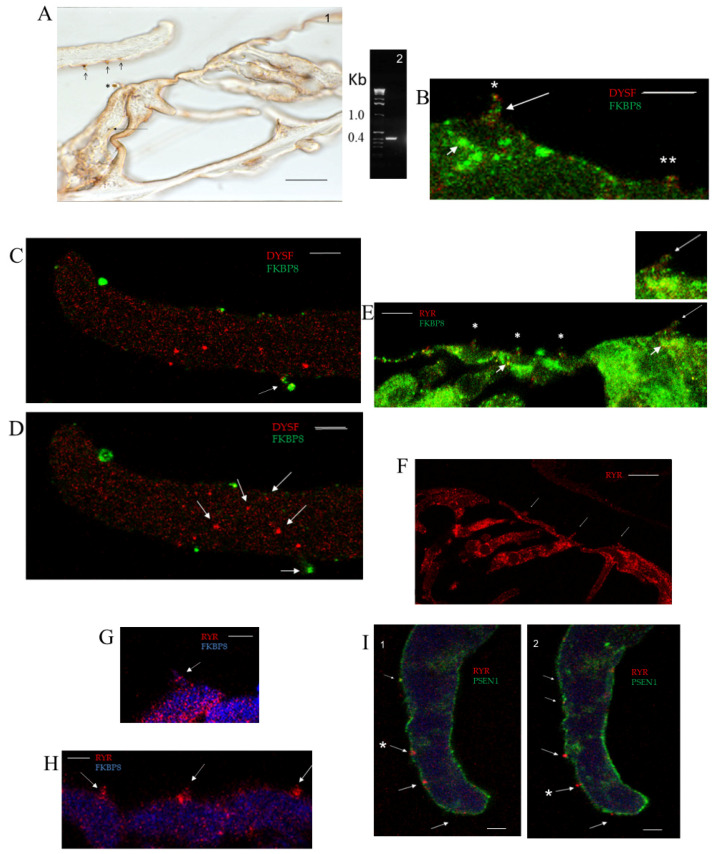
(**A1**) Dysferlin detected with 3,3′-diaminobenzidine (DAB) chromogen staining in middle turn of rat organ of Corti (OC) (P28) with NCL-Hamlet mouse monoclonal antibody (Leica). Dysferlin immunoreactivity (IR) is observed in inner hair cell (IHC) stereocilia (*) and stereocilia from hair cells that are detached and embedded in the tectorial membrane (TM) (short arrows). IR is also found at the base of the IHC (long arrow); scale bar = 10 µm for (**A1**). (**A2**) Dysferlin C2F cDNA from the OC = 415 bp, negative control, row 3. (**B**) Dysferlin (red) co-localized with FKBP8 (green) at the tip of the IHC stereociliary array (single asterisk); the two proteins were also observed adjacent to each other in the outer hair cell (OHC) stereocilia (double asterisks). A row of FKBP8-positive sites (green), possibly corresponding to tips of individual stereocilia in the array, was observed for the IHC (arrow). IHC subcuticular plate mitochondria were immunopositive for FKBP8 (green; small arrow); scale bar = 5 µm. (**C**) Dysferlin, optical section 10 of middle cochlear turn; dysferlin (red, Hamlet) co-localizing with FKBP8 (green) in detached IHC stereociliary insert (arrow) in tectorial membrane (TM); scale bar = 5 µm for (**C**–**E**). (**D**) Dysferlin optical section 8, same slide as for (**C**); dysferlin (red, Hamlet) co-localizing with FKBP8 (green) in detached IHC stereociliary) insert (small arrow). Diagonal lines of immunoreactivity for dysferlin (arrows, red) in the tectorial membrane of the middle cochlear turn. (**E**) FKBP8 mitochondria outer membrane protein (green), ryanodine receptor (RYR, pan antibody, red); co-localization in stereocilia of IHC (yellow, long arrow; small upper right panel, higher magnification of 1.33 ×) and overlapping in OHC (asterisks). Smaller arrow points to close association of FKBP8 and RYR in IHC and OHC subcuticular plate. (**F**) Ryanodine receptors (RYRs) in the OC (pan antibody, red). Arrows point to expression in hair cell stereocilia; scale bar = 10 µm. (**G**) Z-stack optical section 10. RYR (pan antibody, red) in IHC stereocilia (arrow). Light blue = FKBP8; scale bar = 5 µm. (**H**) Z-stack optical section 11 of same slide as for (**G**). RYR (pan antibody, red) in OHC stereocilia (arrows); scale bar = 2.5 µm. (**I**) Tectorial membrane: (**I1**) Z-stack optical section 10 of same slide as for (**G**), alignment (yellow, small arrow) for IHC stereocilia of ryanodine receptor (pan antibody, red) and PSEN1 (green; ABclonal 2187). RYRs from OHC insertions (red, arrows); scale bar = 5 µm. (**I2**). Z-stack optical section 14 (arrows) of same slide as for (**G**) of OHC stereocilia ryanodine receptor inserted in tectorial membrane. Note doublet (arrow + asterisk). RYR receptor is out of focal plane for TM IHC insertion sites (small arrows), differentiating between optical layer for IHC and OHC stereocilia insertions.

Dysferlin, a member of the ferlin protein family that mediates membrane repair in mechanosensory myofibrils, was found to be expressed in the mechanosensory OC (rat) with two highly characterized primary antibodies (Figure 1(A1)), consistent with mRNA expression (Figure 1(A2)). Immunoreactivity with the “Hamlet” antibody (NCL-Hamlet mouse monoclonal for dysferlin targeting synthetic peptide aa 1999–2016 of human dysferlin and crossing to rat dysferlin [2], Leica, Buffalo Grove, IL, USA) (Figure 1(A1)) and “Romeo” anti-dysferlin monoclonal antibody (ABclonal Technology, Woburn, MA, USA, <1:500, Figure 8B) both supported the notion that the dysferlin protein is expressed in the OC. Determination of the relative juxtaposition of the tectorial membrane and hair cell stereocilia expression of protein was enabled by the use of mid-modiolar sections of the middle and apical cochlear turns, as opposed to surface preparations at a 90° reflect-ion. With these caveats, dysferlin immunoreactivity with diaminobenzidine immunostaining was localized to detached hair cell stereociliary arrays inserted in the tectorial membrane (Figure 1(A1)). Immunoreactivity was observed for the inner hair cell (IHC), both in its stereociliary array and at its base (Figure 1(A1), long arrow), with the latter being the site of hair cell transmitter release and otoferlin-mediated transmitter release [20]. Confocal Z-stack immunofluorescence confirmed the localizations of dysferlin in hair cell stereocilia (Figure 1B) and corresponding stereociliary insert positions, both IHC (Figure 1C,D) and outer hair cell (OHC), on the TM (Figure 3(A1,A2); see Figure 1(I1,I2) for discrimination between IHC and OHC stereociliary insert positions). Curiously, lines of dysferlin immunofluorescence were also observed in the tectorial membrane (Figure 1C,D), co-localizing with FKBP8-positive stereociliary array inserts at an IHC position.

FKBP8, a mitochondrial outer membrane marker protein (also representing an immunophilin and m-TOR inhibitor) that directly interacts with dysferlin (K_D_ = 2.4 ± 0.3 × 10^−8^ M), positively regulated by calcium in surface plasmon resonance (SPR) analysis [1], is pivotal in the dysferlin protein–protein interaction (PPI) pathway targeting ryanodine receptors. FKBP8 was originally immunolocalized in the rodent to the tectorial membrane at P6 [5], now extended to hair cells in the adult. In addition to labeling the hair cell stereocilia (Figure 1B), as a marker protein for the mitochondrial outer membrane, FKBP8 immunolocalized to the subcuticular plates of the IHC (Figure 1B,E) and OHC (Figure 1E), being mitochondria-enriched sites with circular mitochondrial figures (Figure 5G). Immunofluorescence for FKBP8 co-localized with immunofluorescence for ryanodine receptors detected with a pan antibody (not distinguishing between ryanodine receptor subtypes) in the stereocilia of the IHC (see insert in Figure 1E) with the immunofluorescence of FKBP8 and RYRs overlapping in OHC stereocilia (Figure 1E). FKBP8 would have a role in both APP and TAU AD pathways [21,22], respectively.

**Figure 2:**
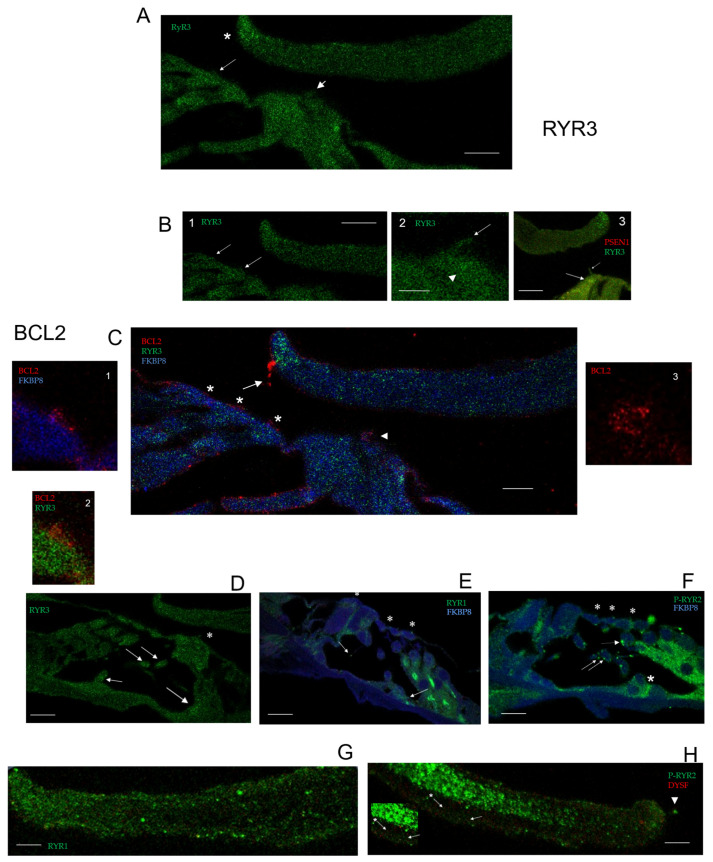
(**A**) Optical position 4 of RYR3 (green) in tip of tectorial membrane (asterisk) and in stereocilia of OHC (arrow) and IHC (arrowhead) of middle cochlear turn; scale bar = 15 µm. (**B1**) Optical position 4, same slide as in (**A**) RYR3 in OHC stereocilia (arrows); scale bar = 15 µm. (**B2**) RYR3 in IHC stereocilia (arrow); RYR3 in subcuticular plate (arrowhead); scale bar = 5 µm. (**B3**) Overlap of PSEN1 IR (red) with RYR3 IR (green) in IHC stereocilia (short arrow) and subcuticular plate (arrow); scale bar = 10.7 µm. (**C**) BCL2 (red, arrow) localized at the distal TM edge continuous with the third-row OHC and in close association with the RYR3-positive tip (green) of the TM. IHC stereocilia (arrowhead) and OHC stereocilia (asterisks) are BCL2-positive (red); scale bar = 5 µm. (**C1**) BCL2 (red) in first row OHC stereocilia with FKBP8 (light blue, magnification 1.5× of (**C**)) and (**C2**) with RYR3 (green, 1.5× of (**C**)). (**C3**) BCL2 (red) in bent-over IHC stereocilia (magnification 3× of (**C**)). The pattern speaks to specificity. (**D**) RYR3 (green) in IHC stereocilia, (asterisk). OHC afferent (shorter arrow) and efferent (medium and long arrows) pathways—compare signal vs. background scale bar = 10 µm. (**E**) Ryanodine receptor 1 (RYR1, green) vs. dark blue background in OC—see evidence for RYR1 in OHC afferent and efferent nerve pathways (arrow and mini-arrow, respectively) and in IHC and OHC stereocilia (asterisks). (**F**) RYR2 OHC efferent (arrows) and afferents (large asterisk) reflect phospho (P)-RYR2 immunoreactivity (green). Hair cell stereocilia were immunoreactive-positive for P-RYR2 (small asterisks); scale bar = 10 µm for (**E**,**F**). (**G**) RYR1 (green) in TM; scale bar = 5 µm. (**H**) P-RYR2 (green) and dysferlin (red) in TM; arrowhead points to P-RYR2 hanging from the tip of the tectorial membrane. Arrow points to diagonal line of P-RYR2 reaching from core to edge of TM closest to HCs. Arrow plus asterisk points to faint diagonal line of dysferlin (red) puncta; scale bar = 5 µm; see inset—1.4 × magnification.

Regarding dysferlin pathway BCL2 and ryanodine receptors, Z-stack confocal immunofluorescence of the cochlear middle/apical turns indicates hair cell stereocilia and tectorial membrane localizations. Ryanodine receptors have multi-site presence in the OC (Figure 1F), associated with both hair cell innervation (Figure 1F) and IHC and OHC stereocilia (Figure 1G,H). RYR immunoreactivity with the pan antibody was found at a site on the tectorial membrane (TM), corresponding to an IHC insert site spatially removed from OHC stereocilia insert sites differentiated in the Z-stack position (Figure 1(I1,I2)) (optical positions 10 and 14), providing a map of hair cell stereociliary insert positions on the tectorial membrane, given the difference in the vertical distance of IHC and OHC inserts perpendicular to optical sections. It was the close association of PSEN1 and RYR (pan antibody) at the IHC stereociliary insert position and the recognized interaction of PSEN1 with RYR3 [23] that led to the use of an antibody specific to RYR3 and the localization of RYR3 in both IHC and OHC stereociliary arrays (Figure 2A,B) and the surprisingly elevated, enhanced concentration of RYR3 at the tip of the tectorial membrane (Figure 2A,B1,B3).

*BCL2* directly interacts with both FKBP8 [6,7] and ryanodine receptors [8]; thus, the dysferlin protein–protein interaction pathway, with FKBP8 as an intermediate, would regulate calcium. BCL2 was found, in particular, in the OC, localized at the distal TM edge continuous with the third-row OHC insertion, close to the RYR3-positive tip of the tectorial membrane (Figure 2A). BCL2 was immunolocalized to IHC and OHC stereocilia arrays (magnifications; Figure 2(C1–C3)) with individual stereocilia resolution.

**Figure 3:**
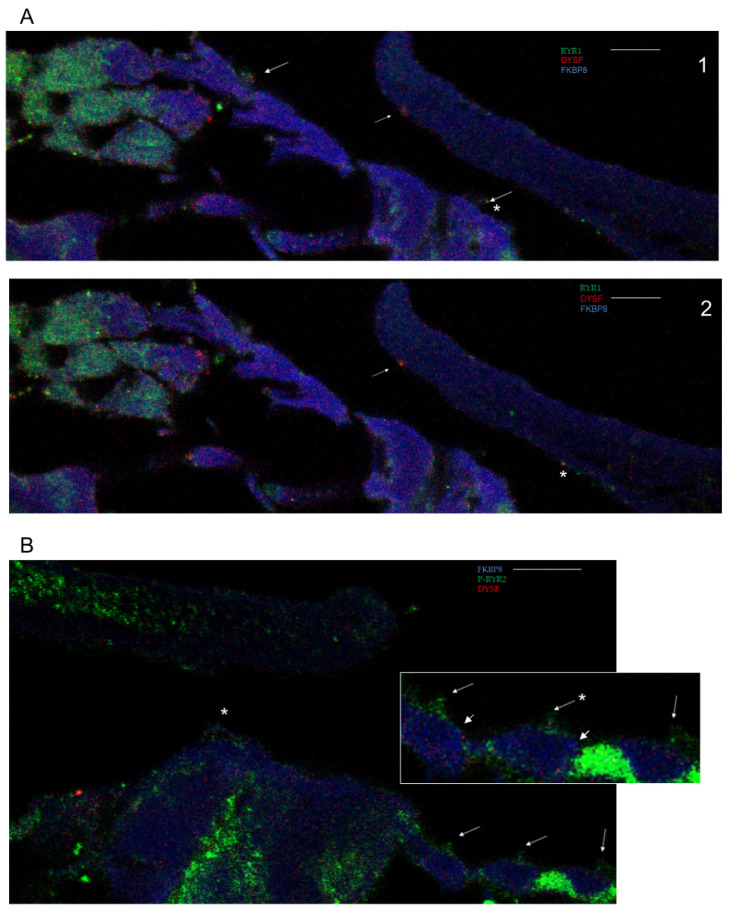
(**A**) Ryanodine receptor 1 (RYR1) and dysferlin in OC. (**A1**) RYR1 (green) and dysferlin (red) in hair cell stereocilia (long arrows) and their IR overlap (yellow) in IHC stereocilia (asterisk). Dysferlin on TM at OHC stereocilia insert site (short arrow); scale bar = 5 µm. (**A2**). Dysferlin (red) co-localized with RYR1 (green) on TM at IHC stereocilia insert position (asterisk). Dysferlin hanging onto TM at OHC stereocilia insert position (short arrow); scale bar = 5 µm. (**B**) Ryanodine receptor 2 (P-RYR2; green) and dysferlin (red). IHC stereocilia were immunoreactive-positive for P-RYR2 (asterisk). Insert magnification indicates P-RYR2 on OHC stereocilia (arrows) and overlap with dysferlin (arrow + asterisk); scale bar = 5 µm; magnified inserts = 1.7 ×. FKBP8 expression is indicated by lighter blue at apical hair cell sites overlaying dark blue background (pair of short arrows on magnified inserts).

Ryanodine receptor subtype specificity in the OC contributes to pan RYR expression. Confocal Z-stack immunofluorescence to RYR1 and phospho-RYR2-specific antibodies indicated respective isoform contributions to RYR expression in IHC and OHC stereocilia (Figure 2E,F), as well as in OHC efferent and afferent pathways and the TM (Figure 2). RYR1 co-localized with dysferlin in stereocilia (Figure 3(A1)) and RYR1 immunoreactivity (IR) was also found in the “core” of the tectorial membrane (Figure 2G). P-RYR2 IR was found in stereocilia (Figure 3B) and the TMextending in diagonal lines to the lower edge of the TM nearest to the hair cells (Figure 2H, P-RYR2 arrow; dysferlin arrow plus asterisk; insert 1.4×).

**Figure 4:**
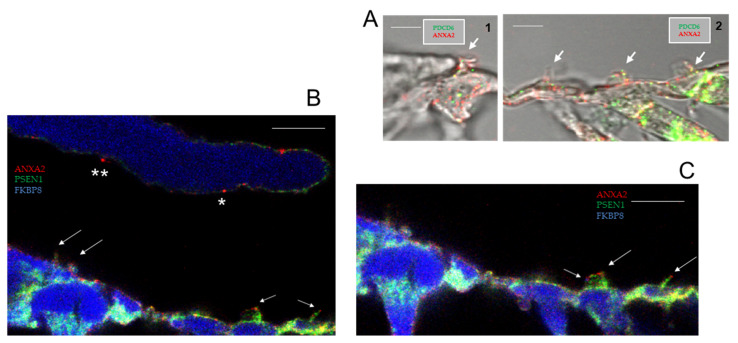
(**A**) Expression of PDCD6 (green), recognized as a new gene in hearing loss [22], compared with dysferlin (red) or annexin A2 (red) in OC, detected with Z-stack (1 µm sections) confocal immunofluorescence. (**A1**) PDCD6 (green) and annexin A2 (red) in IHC stereocilia of the apical cochlear turn. (**A2**) PDCD6 co-localizes with annexin A2 (in yellow) in the stereocilia of OHC (arrows), consistent with tight protein–protein interaction (PPI) indicated by SPR; scale bar for (**A1**, **A2**) = 2.5 µm. (**B**) Z-stack optical section 8. Annexin A2 (red) immunolocalized to stereocilia of IHC (arrows) and OHC (short arrows) with annexin A2 insertion sites observed in the tectorial membrane corresponding to IHC stereocilia inserts (**) and OHC stereocilia insert (*). PSEN1 (green; ABclonal A2187) immunolocalized to stereocilia of IHC and OHC; scale bar = 5 µm. (**C**) Z-stack optical section 7 of same slide as in (**B**). Stereociliary arrays for OHC (arrows) indicate annexin A2 (red) at stereociliary tips in mid-modiolar section (middle turn), followed by PSEN1 (green) and FKBP8 (blue) (short arrow); scale bar = 5 µm.

Annexin A2 is prominent in the dysferlin PPI pathway, directly interacting with both dysferlin and PDCD6 [1], a hearing loss protein [24]. Annexin A2 co-localizes with PDCD6 in OHC stereocilia (Figure 4(A2), yellow) and is found at the tip of OHC stereocilia relative to ordered sites of other stereociliary proteins (PSEN1 and FKBP8) (Figure 4B,C). Annexin A2-positive stereociliary inserts were observed in the TM, corresponding to IHC and OHC insert positions (Figure 4B).

### 2.2. Wildtype Expression of Alzheimer’s Disease Proteins in the Organ of Corti

The wildtype expression of proteins that, when mutated, epitomize Alzheimer’s disease (AD) is not a given for the cochlea. While the significant expression of amyloid precursor protein (APP) has been reported in brain control tissue, enhanced in BACE inhibitor NB-360-treated mice [15], little amyloid-β was detected in wildtype cochlea with enhanced immunodetection, providing the clear microscopic localization of APP in a transgenic mouse model of AD [11]. The presumption of the specific localization and molecular function of AD proteins in the wildtype was considered in the present investigation.

**Figure 5:**
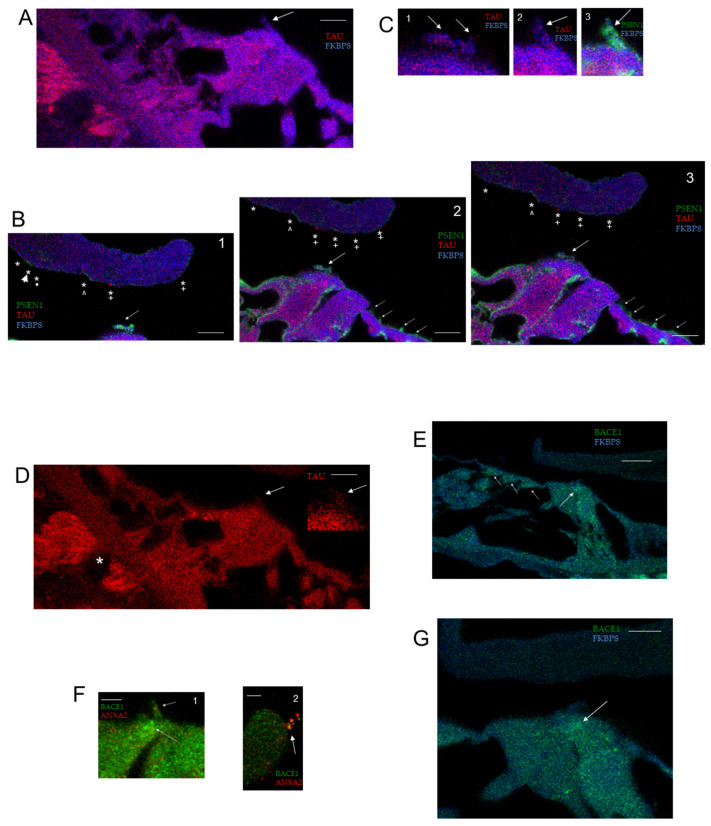

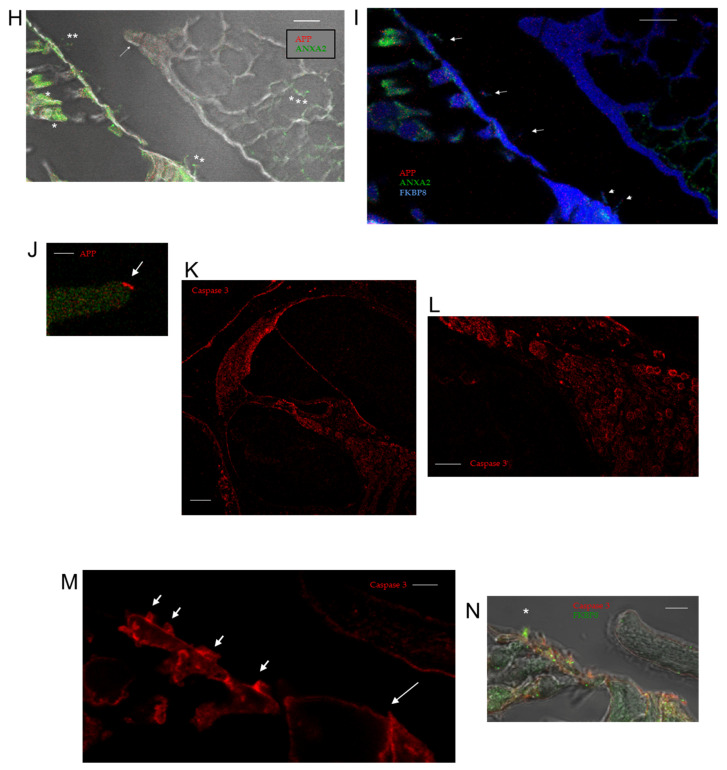
Alzheimer’s disease wildtype proteins in the OC: TAU, PSEN1, BACE1, APP, and caspase 3. (**A**) Z-stack optical section 17: TAU (red) and FKBP8 (blue) in nerve fibers approaching habenula perforata (magenta, also see asterisk D5); TAU and FKBP8 in IHC stereocilia (arrow); scale bar = 5 µm. (**B1**) Z-stack optical section 13: PSEN1 (green; ABclonal A2187) and FKBP8 (blue) are closely localized in stereocilia arrays of IHC (arrow). Stereociliary insertions on the tectorial membrane are designated: asterisk indicates FKBP8 IHC insert site in the TM; arrowhead with asterisk indicates IHC stereocilia insert site on TM positive for PSEN1 (green) hanging on the TM. Asterisk plus inverted v indicates another IHC insertion site position for PSEN1. OHC insertion sites included one positive for TAU (red, asterisk and + sign). (**B2**) Z-stack optical section 14: PSEN1 (green) and FKBP8 (blue) are localized to stereocilia of IHC (arrow) and OHC stereocilia (shorter arrows). Stereociliary insertion sites are observed in tectorial membrane as designated in (**B1**). OHC insertion sites were positive for TAU (red, asterisk and + sign). (**B3**) Z-stack optical section 15: TAU (red) immunolocalized close to FKBP8 (blue) in insertions (*) on tectorial membrane designated as in (**B1**); scale bar for (**B1**–**B3**) = 5 µm. (**C**) Z-stack (**C1**) optical section 13: TAU (red) localized close to FKBP8 (blue) for IHC stereociliary arrays (arrows) at 2.27x magnification of (**B1**). (**C2**) Optical section 17 (magnified, arrow) and (**C3**) FKBP8 (blue) and PSEN1 (green, ABclonal 2187; arrow) in IHC stereocilia; magnification = 2.25 × of (**A**) for (**C2**,**C3**). (**D**) TAU in OC with TAU immunopositive nerve fibers entering the habenula perforata (*). Arrow points to IHC stereocilia with diagonal immunoreactivity pattern, predictive of individual stereocilia in the array; scale bar = 5 µm; insert 2 ×. (**E**) BACE1 (green) and FKBP8 (blue) in OC overview. Overlapping immunoreactivity observed in OHC subcuticular plate (short arrows) and IHC subcuticular plate (long arrow); scale bar = 10 µm. (**F1**) BACE1 (green, short arrow) immunoreactivity does overlap that of annexin A2 (red) in IHC stereocilia (yellow–orange). BACE1 is also seen in the IHC subcuticular plate (yellow–green, long arrow); scale bar = 2.5 µm. (**F2**) BACE1 immunoreactivity overlaps that of annexin A2 in fragment hanging from TM tip; scale bar = 2.5 µm. (**G**) Overlapping expression of FKBP8 (blue) and BACE1 (green) was observed in IHC subcuticular plate circular mitochondria sites (arrow); scale bar = 5 µm. (**H**) APP (red) + annexin A2 (green) + brightfield. Overlap (yellow) seen in OHC innervation * and occasionally in HC stereocilia **. Note APP in TM tip (small arrow) and annexin A2 in TM body ***; scale bar = 10 µm. (**I**) APP (red), annexin A2 (green), FKBP8 (light blue against dark blue background) in apical turn. Short arrows indicate APP in OHC stereocilia; arrowheads indicate APP in stereocilia of IHC; scale bar = 10 µm. (**J**) APP (red, arrow) at tip of TM; scale bar = 5 µm. (**K**) Caspase 3 in the cochlea; scale bar = 10 µm and (**L**) in the SG; scale bar = 5 µm. (**M**) Caspase 3 in OHC stereocilia (small arrows) and IHC stereocilia (long arrow); scale bar = 2.5 µm. (**N**) Caspase 3 (red) immunoreactivity overlaps that of FKBP8 (green) in OHC stereocilia (*) in brightfield image; scale bar = 5 µm.

BACE1, the β-secretase protease converting amyloid precursor protein (APP) to toxic amyloid-β peptides in AD, was previously determined to be required for normal hearing with neural targets in wildtype cochlea [15]. We immunolocalized BACE1 protein additionally to the subcuticular plate of both the IHC (Figure 5(F1,E,G)) and OHC (Figure 5E). BACE1 co-localized with FKBP8 in the subcuticular plate of the IHC (Figure 5E,G) and further co-localized with annexin A2 in both the IHC stereocilia (Figure 5(F1)) and a fragment/OHC stereocilia insert attached to the tip of the TM (Figure 5(F2)).

Presenilin 1 (PSEN1), one component of ɣ-secretase, the aspartyl protease cleaving APP to produce Aβ peptide in AD, is expressed in the wildtype OC (Figure 5(B1–B3,C3)). PSEN1 competes with FKBP8 to target BCL2 [25]. Mutations in the psen1 gene are cited as the most common cause of early-onset, familial Alzheimer’s disease (FAD). Presenilin1 co-localized with RYR in the IHC stereocilia insert position on the TM (pan RYR antibody, Figure 1(I1)), as well as specifically with RYR3 (Figure 2(B3)). PSEN1 was closely associated with FKBP8 in IHC stereocilia (Figure 5(B1–B3)) and with TAU (Figure 5(C1)). The OHC stereocilia were immunopositive for PSEN1, alternating with annexin A2 (Figure 4B,C). These results are in line with an apparently inhibitory role of PSEN1 in tubulin-associated unit (TAU) phosphorylation and secretion [26], competition between PSEN1 and ryanodine receptors in interacting with BCL2, and a recognized direct interaction of PSEN1 and FKBP8 [25]. The ɣ-secretase complex includes PSENEN/PEN-2, required for the transmembrane protein processing of APP. PSENEN is a direct binding partner of CNGA3 (yeast two-hybrid [27], unpublished result). CNGA3 is localized in a specific pattern in OHC hair cell stereocilia (Supplementary Results, Figure S1A), possibly reflecting the direct binding of CNGA3 to myosin VIIa and cadherin 23 [27], which would place CNGA3 at the upper end of the stereociliary tip link. CNGA3 is coupled with CNGB1 in hair cell stereocilia (Supplementary Materials, Figure S1B,C).

TAU maintains the stability of microtubules and, when hyperphosphorylated, forms helical filaments—a characteristic of AD—previously localized to nerve fibers, hypothesized to be afferent, beneath the IHC and OHC and in the osseous spiral lamina [28]. Subcuticular plate localization was found in vestibular type II hair cells [29]. We obtained evidence of sites of expression in the OC, in addition to the nerve fibers of the osseous spiral lamina (Figure 5A–D). TAU immunoreactivity was found at specific sites on the TM, consistent with stereociliary insert sites (Figure 5(B1–B3)). FKBP8 transport and its role in mitophagy are considered coupled to TAU phosphorylation [22], another link to TAU hyperphosphorylation in AD. TAU immunoreactivity overlapped that of FKBP8 in nerve fibers approaching the habenula perforata (Figure 5A also see asterisk in Figure 5D for TAU-positive nerve fibers). Anti-inflammatory compounds such as FKBP8 block TAU pathology [30].

Regarding APP, FKBP8 directly interacts with amyloid beta precursor protein binding family B member 1 (APBB1) [NCBI Gene], which in turn interacts with AD amyloid precursor protein (APP), raising the possibility of ameliorative manipulation with FKBP8 in Alzheimer’s disease. APP immunoreactivity within the OC appeared adjacent to/overlapping the immunoreactivity of annexin A2 (Figure 5H,I). APP immunoreactivity was observed at the tip of the TM (Figure 5J).

Caspase 3 is a cysteine-aspartic acid protease involved in the cleavage of amyloid-beta 4A precursor protein, which is associated with neuronal death in Alzheimer’s disease. Its activity is inhibited by the Aβ42 monomer [31]. It interacts with PSEN1, competing with FKBP8 for BCL2, and also interacts with PDE5A/6C and, as a consequence, CNGA3. Caspase 3 is expressed in the OC (Figure 5K) and SG (Figure 5L). Caspase 3 is concentrated in both OHC and IHC stereocilia (Figure 5M), where it co-localizes with FKBP8 (Figure 5N *). FKBP38 protects BCL2 from caspase-dependent degradation [32].

### 2.3. Molecular Targeting of Alzheimer’s Disease-Associated Proteins in the Spiral Ganglion and Cochlear Neural Components by Dysferlin Protein–Protein Interactions

**Figure 6:**
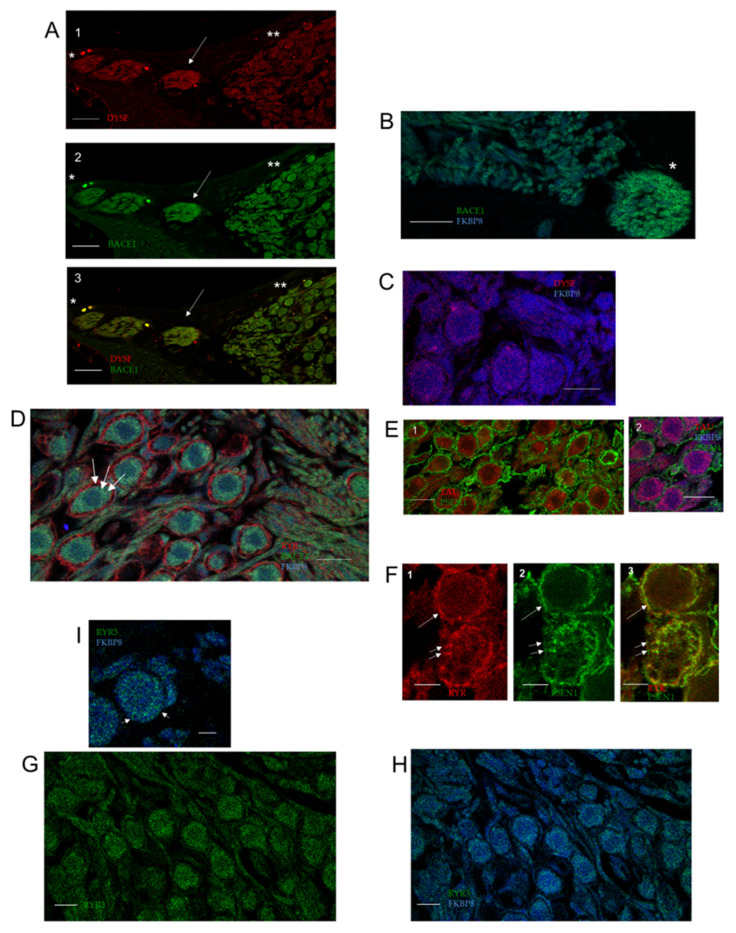
Dysferlin pathway proteins and wildtype AD proteins in the SG and neural elements. (**A1**) Low-magnification image of dysferlin (Hamlet, red, long arrow); (**A2**) BACE1 (green); (**A3**) dysferlin and BACE1 were in close association in nerve fibers approaching the habenula perforata (asterisk) and in the SG (two asterisks); scale bar = 5 µm for (**A1**–**A3**). (**B**) Low-magnification image of BACE1 (green) and FKBP8 (blue), which were closely aligned in nerve fibers (*), approaching the habenula perforata (asterisk); scale bar = 20 µm. (**C**) Dysferlin (red, Hamlet) and FKBP8 (light blue) on SG type I cell bodies; scale bar = 10 µm. (**D**) BACE1 (green), FKBP8 (blue) RYR (red, pan antibody). Arrows points to close association of FKBP8 (blue) and BACE1 (green) in circular mitochondria-like structures in type I cell bodies of the SG; scale bar = 10 µm. (**E1**) PSEN1 (green; ABclonal 2187) on plasma membranes of type I cell bodies in SG and TAU (red) in interiors of type I cell bodies; scale bar = 10 µm. (**E2**) TAU (red) aligned with FKBP8 (blue) in interiors of type I SG cell bodies but not on plasma membranes with PSEN1; scale bar = 10 µm. (**F1**) RYR receptor (pan antibody, red) and (**F2**) PSEN1 (green; ABclonal 2187) on plasma membranes of type I cell bodies (arrows) extending inward (short arrows) and (**F3**) in close association with RYR (yellow); scale bar = 5 µm for (**F1**–**F3**). The Manders coefficient was 0.97 (Volocity 7.0.0 software). (**G**) RYR3 (green). (**H**) RYR3 aligned with FKBP8; scale bar = 10 µm for (**G**,**H**). (**I**) RYR3 (green) and FKBP8 (blue); scale bar = 5 µm. Lines of RYR3 IR extend inward from the plasma membrane (small arrows), similarly to IR with the pan RYR antibody (**F1**).

Evidence for separate groups of protein clusters was replicated in the SG and nerve fibers approaching the habenula perforata. Dysferlin immunoreactivity overlapped that of BACE1 in the nerve fibers approaching the habenula perforata and osseous spiral lamina (Figure 6(A1–A3)) and likewise closely aligned with FKBP8 (Figure 6B). Within the SG, dysferlin (Figure 6C) and BACE1 IR clearly overlapped with FKBP8 IR, with the latter, BACE1 and FKBP8, in circular mitochondrial figures (Figure 6D, white arrows), distinguished from ryanodine receptor immunoreactivity (RYR pan antibody) on the membrane surface (Figure 6D). TAU also was in close association with FKBP8 in the interiors of the type I cell bodies (Figure 6(E1,E2)), not aligning with PSEN1 on the cell membranes of type I SG cell bodies. Lines of RYR, detected with a pan antibody and overlapping with PSEN1 IR, appeared to enter the interior of the cell body (Figure 6(F1–F3)) (with evidence obtained for statistical co-localization).

**Figure 7:**
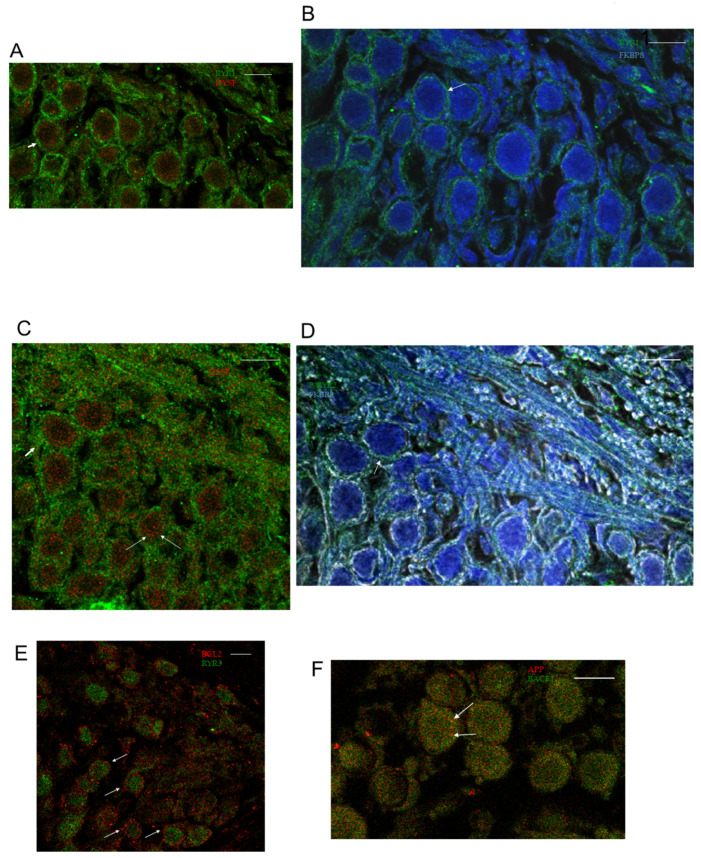
Dysferlin pathway proteins and wildtype AD proteins in the SG and neural elements, continued. (**A**) Ryanodine receptor 1 (green) on cell membrane extending inward (arrow) and dysferlin (red) in type 1 cell bodies of SG; scale bar = 10 µm. (**B**) RYR1 (green) on cell membrane and FKBP8 (light blue) in type I cell bodies of SG. Note circular FKBP8 figures (mitochondria, arrow); scale bar = 10 µm. (**C**) Ryanodine receptor 2 (green) on cell membrane extending inward (short arrow) and rows of dysferlin puncta (red) in type I cell bodies of SG (long arrows); scale bar = 10 µm. (**D**) P-RYR2 (green, arrow) on cell membrane and FKBP8 (blue) in type I cell bodies of SG plus brightfield; scale bar = 10 µm. (**E**) BCL2 (red) and RYR3 (green). BCL2 was localized to outer membranes of SG type I cell bodies (arrows); scale bar = 10 µm. (**F**) APP (red) and BACE1 (green) co-aggregated (yellow, arrows) in the interiors of SG type I cell bodies; scale bar = 10 µm.

A portion of the overall RYR immunofluorescence of the type I SG cell bodies observed with the pan antibody appeared to be contributed by RYR3 (Figure 6G–I) and, in particular, associated with lines of RYR immunofluorescence extending from the outer membrane to the interior of the cell body (Figure 6I). Unlike RYR3, RYR1 (Figure 7A,B) and P-RYR2 (Figure 7C,D) were primarily confined to the outer cell membranes of the type I cell bodies of the SG segregated from the immunoreactivity of dysferlin and FKBP8. APP immunoreactivity overlapped with BACE1 in the interiors of the type I cell bodies (Figure 7F). BCL2 (Figure 7E) and caspase 3 (Figure 5L) appeared on the outer cell membranes of the cell bodies of type I SG neurons and, for BCL2, also type II SG fibers (the latter not illustrated).

### 2.4. Dysferlin Protein–Protein Interactions Altered in Dysferlinopathy, Potentially Ameliorative in Alzheimer’s Disease

**Figure 8:**
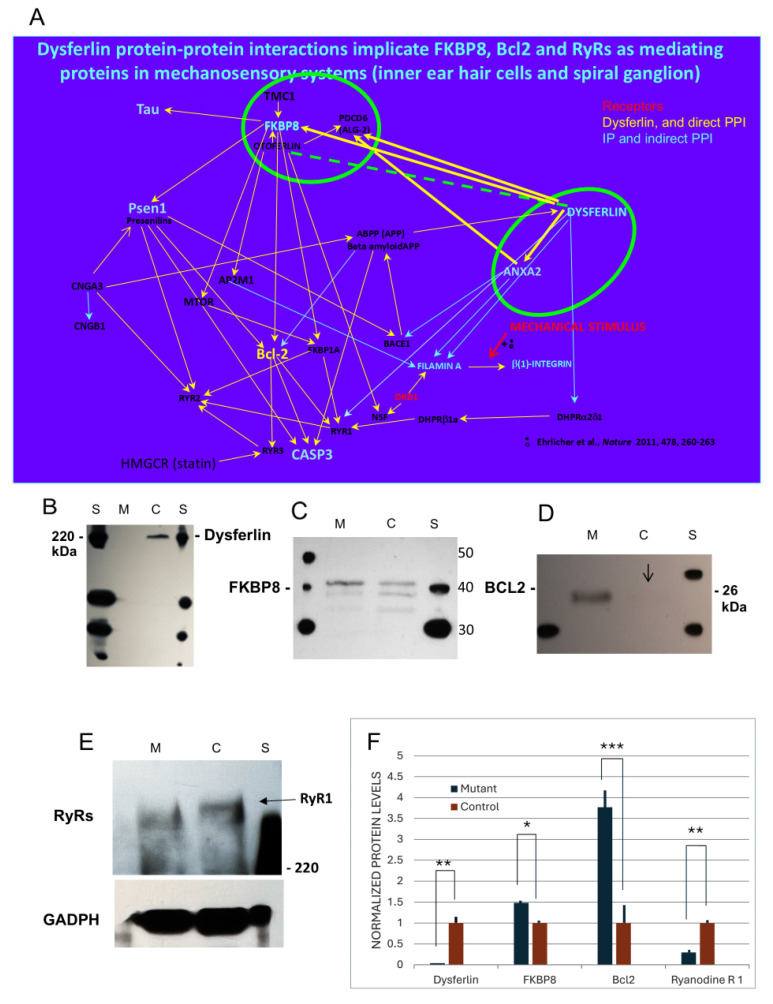
Dysferlin protein–protein interaction pathways and wildtype AD proteins. (**A**) Dysferlin protein–protein interaction pathways. (**B**,**F**) Western blot of dysferlin control (**C**) compared to dysferlinopathy mutant (M) (mouse gastrocnemius) detected with the “Romeo” antibody. Dysferlin is absent/close to absent in the dysferlinopathy mutant (M) compared to control (**C**) according to Student’s *t*-test unpaired and paired variate analysis, the latter relative to paired adjacent lane control values (and relative to total protein) (GraphPad): unpaired two-tailed *p*-value = 0.0006 (**); paired *t*-test two-tailed *p*-value = 0.0054. (**C**,**F**) The FKBP8-positive band at 43 kDa is elevated in the dysferlinopathy mutant (M) compared to control (**C**): unpaired two-tailed *p*-value = 0.0327 (*); paired *t*-test two-tailed *p*-value = 0.0026. (**D**,**F**) BCL2 is highly elevated in dysferlinopathy mutant (M) compared to control (**C**) in gastrocnemius muscle: unpaired two-tailed *p*-value = 0.0007 (***); paired *t*-test two-tailed *p*-value = 0.0001. (**E**,**F**) Western blots for ryanodine receptor(s) RYR1 in control (**C**) and mutant (M) gastrocnemius. Dysferlinopathy mutant tissue (M) had reduced levels of RYR1: unpaired two-tailed *p*-value = 0.0011 (**); paired *t*-test two-tailed *p*-value = 0.0082. (**F**) Western blot bar graph for mutant vs. control gastrocnemius normalized to total protein. Dysferlin, *n* = 5; FKBP8, *n* = 4; BCL2, *n* = 7; RYR1, *n* = 4.

Western blot analysis indicate that, in dysferlinopathic male mice (BlaJ—B6.A-Dysf prmd) (7–9 months), dysferlin expression was eliminated (Figure 8B,F) in the gastrocnemius muscle of the mutant (M) compared with the control (C) mouse. FKBP8 at 43 kDa was elevated in the absence of dysferlin (Figure 8C,F), and BCL2 was significantly elevated (Figure 8D,F), coupled with the essential elimination of RYR1 (Figure 8E,F). The alterations in the expression of proteins comprising the dysferlin PPI in dysferlinopathy would be consistent with FKBP8/BCL2 amelioration in Alzheimer’s disease or the reversal of BCL2 reduction linked to RYR receptor activation characterizing AD.

## 3. Discussion

### 3.1. Implications of Dysferlin Expression in the Organ of Corti: Hair Cell Stereocilia and Inserts in the Tectorial Membrane

In the present investigation, dysferlin was clearly localized to hair cell stereociliary arrays. Unexpectedly, hair cell stereociliary insertions into the tectorial membrane in the mid-modiolar sections of apical/middle cochlear turns displayed immunoreactivity, raising the possibility of a molecular mechanism to alter tectorial membrane Ca^2+^, suggested to regulate hair cell mechanotransduction near the stereocilia [9] and controlling hearing sensitivity [33]. A source of tectorial membrane Ca^2+^ contributing to hair cell mechanotransduction has not been previously identified, although both OHC and IHC stereocilia are recognized as inserting into the tectorial membrane and connecting to granular structures [34]. Further, our studies suggested a tectorial presence of dysferlin, differentiated from the stereocilia insertions.

We obtained evidence for the basal localization of dysferlin in IHC as well, which would correspond to the position of otoferlin, another closely related ferlin having a role in IHC exocytosis [20]. Otoferlin, when mutated, underlies the non-syndromic deafness DFNB9, and so the question is whether dysferlin mutation could give rise to deafness. The dysferlin gene locus 2p13.2 has not been identified by itself as targeted in deafness. However, it is in close proximity to ALSM1, underlying Alström syndrome deafness, on chromosome 2p13.1, and the two genes have been linked in human deafness [35]. Z-stack confocal fluorescence microscopy also confirmed the hair cell expression of proteins comprising the dysferlin PPI pathway, supporting dysferlin functionality.

### 3.2. Expression of Wildtype Alzheimer’s Disease Proteins in the Organ of Corti

Given that dysferlin has been implicated as a molecular marker for AD, accumulating in brain plaques [36], we additionally examined the localizations of BACE1, APP, PSEN1, and TAU, representing proteins that, when mutated, facilitate AD. Wildtype PSEN1 IR overlapped RYR IR (pan antibody) on the tectorial membrane at the IHC stereocilia insert sites and was also found in the OHC stereocilia, alternating with annexin A2. PSEN1 has been cited as regulating Ca^2+^ homeostasis via RYR3 [37], and PSEN1 specifically interacts with RYR3 [23], which we have now localized to the IHC stereocilia. In a separate publication, it is stated that “PS1/2 directly binds FKBP8, forming a macromolecular complex with BCL2” [25], which, by inference, may include amyloid beta precursor protein binding family B member (APBB1), which itself directly interacts with both APP and FKBP8 [38]. PS1/2 is thought to promote the degradation of a complex of BCL2, PSEN1, and FKBP8, with BCL2 sequestering these proteins to ER/Golgi compartments, thereby inhibiting the FKBP8-mediated mitochondrial targeting of BCL2. The FAD-linked PS1/2 mutants were hypothesized to enhance pro-apoptotic activity by causing a more efficient reduction in mitochondrial BCL2 than did wildtype PS1/2 [25]. These results suggest a novel molecular mechanism for the regulation of mitochondria-mediated apoptosis by competition between PS1/2 and FKBP8 for the subcellular targeting of BCL2, which would have consequential effects on RYRs.

Evidence for the association of PSEN1/PSEN2 and hair cell proteins also exists. The molecular complex of the secretase forming APP from β-amyloid includes PSENEN/PEN-2, a direct binding partner of CNGA3, according to a yeast two-hybrid analysis ([25]; unpublished result). CNGA3 and CNGB1 immunofluorescence co-localized in hair cell stereocilia and was found at insert sites on the tectorial membrane (Supplementary Materials).

CNGA3 also directly interacts with LRP1, an apolipoprotein E-binding protein, according to a yeast two-hybrid analysis (an unpublished result from [27]). LRP1 itself interacts with BACE1 and APP, “necessary for alpha 2 macroglobulin-mediated clearance of secreted amyloid precursor protein and beta-amyloid, the main component of amyloid plaques found in an Alzheimer patient” [LRP1, LDL receptor-related protein 1 Homo sapiens (human)—NCBI Gene]. The combined evidence points to CNG channel involvement in AD.

Within the cochlea, the unmutated protein β-secretase BACE1 has been the most extensively investigated of the AD wildtype proteins [15], recognized for its neural distribution. In the present investigation, BACE1 has additionally been localized to the OHC stereociliary TM insert, in close conjunction with annexin A2, another member of the dysferlin PPI pathway. BACE1 aligned with annexin A2 in the IHC stereocilia and subcuticular plate. BACE1 was also closely associated with FKBP8, the mitochondria outer membrane protein, in the subcuticular plate of the cochlear inner hair cell. “Altered mitochondrial homeostasis is considered an early event in AD development” [21,39], with the accumulation of APP C-terminal fragments affecting mitochondrial structure and function. Mitochondrial potentials are also altered in AD [40]. The possibility of FKBP8 redirecting BCL2 and RYR in AD is reiterated.

BCL2 in cochlear hair cells is recognized as ameliorative in reversing aminoglycoside toxicity [41]. Aminoglycosides are thought to enter hair cells via the mechanotransduction channel; thus, the localization of BCL2 to hair cell stereocilia would be consistent with this function. BCL2 interacts with both FKBP8 and ryanodine receptors.

### 3.3. Ryanodine Receptors in Hair Cells and the Tectorial Membrane

Ryanodine receptor expression in cochlear hair cells has long been recognized [42,43,44,45,46]. However, ryanodine receptors have not hitherto been recognized as contributors to the regulation of stereocilia Ca^2+^ or for their appearance in the tectorial membrane, with the latter presumably regulated by the interdental cells of the spiral limbus with their involvement in Ca^2+^ regulation [10]. Ascertaining ryanodine receptor subtype expression in the OC is recognized as highly dependent on primary antibody recognition [47]. With the highly acclaimed pan RYR antibody, we obtained evidence for stereociliary RYR, also previously observed [48], along with specificity for IHC- versus OHC-interacting proteins from the inserts in the tectorial membrane. Then, the issue becomes the subtype identity of the RYRs, since these receptors are noted for their differential properties, localizations, and combinations [49].

In our investigation, we observed more immunoreactivity with the pan RYR antibody in HC stereocilia and the tectorial membrane than attributable to RYR3, suggesting possible contributions from other RYR types. We have determined with yeast two-hybrid analysis that CNGA3 directly interacts with RYR2 ([27], unpublished results), with the latter consequently representing a good candidate, contributing to the overall RYR receptor immunoreactivity. RYR2 upregulation is observed in cyclic nucleotide-gated channel deficiency cone degeneration, implicating an association with CNGA3 [50], and CNGA3 is also observed in hair cell/tectorial membrane locations (Supplementary Materials). RYR2 has been suggested as a direct binding interactor of dysferlin in cardiac hypertrophy [51], representing another possible dysferlin-related mechanism for altering hair cell Ca^2+^, in addition to the dysferlin PPI pathway, not previously recognized [52]. Whether the tubular membrane localization could be related to the calcium ducts in the tectorial membrane [34] and the appearance of “lines” of dysferlin immunoreactivity in the TM, and the possible co-localization of dysferlin in the TM with RYR2, remains an unanswered question.

### 3.4. Ryanodine Receptors in Neural Pathways of the Organ of Corti

Confocal microscopic localization has suggested that RYRs are concentrated in the SG and in emanating afferent pathways with the highest expression of RYR3 [53]. OHC efferent localization of RYRs (RYR1) has also been implicated, without obviating the participation of other RYRs [47]. In the present investigation, OHC and IHC afferents were immunopositive for RYR3, RYR1, and P-RYR2. Further, immunoreactivity for P-RYR2, in particular, was associated with olivocochlear medial (crossing) efferent fibers.

### 3.5. Alzheimer’s Disease-Associated Proteins in the Spiral Ganglion and Cochlear Neural Components

Two types of protein clusters related to AD are present in type I SG cell bodies and afferent nerve fibers that would enable AD mutation-linked hearing deficits. The first, which would have input from dysferlin and dysferlin PPI pathways, are mitochondrial targets, anatomically defined by the localization of the mitochondrial outer membrane marker protein, FKBP8, along with AD proteins BACE1 and TAU. A second protein complex on the cell body plasma membrane includes the ryanodine receptors that would regulate Ca^2+^ and AD proteins PSEN1, BCL2, and caspase 3. Images were observed of RYR along with PSEN1 internalizing towards FKBP8 outer membrane mitochondrial targets co-localizing with TAU, BACE1, and APP. Further, dysferlin itself was structurally arranged in rows within the SG cell bodies, suggesting internally projecting mitochondrial platforms observed in skeletal muscle myofibrils. The possibility of dysferlin reversing the effects of AD Aβ1-42 by blocking autophagy [54] leads to the question of a mitochondrial translational mechanism and the PPI that underlies it.

### 3.6. Dysferlin and Ryanodine Receptor Function in Alzheimer’s Disease

Multiple reports suggest that ryanodine receptors are activated in AD, and BCL2 is depressed [55,56,57,58]. In part, the former may be related to the age-related loss of the transcriptional expression of miR-124, derepressing RYR3 expression [59]. Statins such as Rosuvastatin can increase the expression of miR-124-3p, attenuating total TAU serum levels [60], linking the reduction in RYR activity to the amelioration of AD. Ryanodine receptor function is also known to be affected by PSEN1 and its N-terminus cysteine composition [61], as well as by caspase 3 [58].

## 4. Materials and Methods

### 4.1. Analysis of Dysferlin cDNA

Oligonucleotide primers designed with the Accelrys 7.0 software (San Diego, CA, USA) were used to determine the expression of dysferlin C2F in OC cDNA. PCR reactions were carried out in 50 µL reaction volumes containing BD Advantage 2 polymerase mix (BD Biosciences Clontech, San Jose, CA, USA). The temperature profile of the PCR reactions was 95 °C for 3 min, 40 cycles of 95 °C for 45 s, 60 °C for 30 s, and 72 °C for 1.5 min, followed by a 10 min extension at 72 °C. Appropriately sized PCR products were sliced from low-melting-point agarose gels, and the DNA was extracted using a Qiaex II PCR Purification Kit (Qiagen, Valencia, CA, USA) and sequence-verified. Organ of Corti cDNA for the C2F domain of dysferlin was amplified by PCR with primers crossing introns. The predicted Δ = 415 bp (0.415 Kb). Negative controls did not include OC cDNA.

### 4.2. Immunohistochemistry

Immunoreactivity in paraffin-imbedded rat cochleae (Black Agouti ACI rats of mixed gender, processed as described in [18]) was visualized with the avidin–biotin complex peroxidase method (ABC Elite protocol, Vector, Burlingame, CA, USA), with 3,3′-diaminobenzidine (DAB) serving as a chromogen (Bio-Genex, San Ramon, CA, USA). Here, 4–5 µm deparaffinized sections of middle/apical cochlear turns cut in the mid-modiolar plane were sequentially incubated in 0.1% sodium borohydride and 5 mM glycine in phosphate-buffered saline (PBS) for 45 min, 3% H_2_O_2_ in tap water for 5 min, 2% normal serum (corresponding to the species in which the secondary antibody was raised) in PBS, and primary antibody at 4 °C for 12–16 h [2], followed by biotinylated donkey anti-mouse IgG for 30 min at room temperature. Immunostaining with DAB was examined with a Leitz Diaplan microscope (Leitz, Wetzlar, Germany) and photographed with an Olympus OM-4T camera and the negatives digitized at 300–600 dpi. Individual DAB negative controls were obtained with the omission of the primary antibody and inclusion of a secondary antibody, polyclonal donkey anti-mouse IgG or polyclonal donkey anti-rabbit IgG.

### 4.3. Confocal Fluorescence Microscopy

Protein expression in the organ of Corti and spiral ganglion was examined with confocal microscopy immunofluorescence Z-stacks using a Zeiss LSM 780 instrument at 63× magnification with oil and 0.6–1.0 μm slices. Each fluorophore was acquired on a separate track (i.e., a sequential scan), with a sufficient wavelength distance to ensure that there was no overlap. Track one was acquired on Ch1 (415–463 nm), Track 2 was acquired on channel S1(499–552 nm) (Alexa 488-green), and Track 3 was acquired on Ch2 (572–647 nm) (Alexa 568-red). These were three separate detectors on the LSM 780 and ensured that no spectral overlap or bleed through occurred. Laser lines for the Zeiss LSM 780 were 405 nm (diode laser), 458 nm, 488 nm, 514 nm (multiline argon laser), 561 nm, 633 nm (helium-neon laser), and 488–645 nm (tunable white light laser). Detector bandwidths were 415–477 nm (channel 1), 410–695 nm (channel S1), and 645–735 nm (channel 2). The pinhole for channel 1 was 44 µm, that for channel S1 was 52 µm, and that for channel 2 was 81 µm. The pixel dwell time was 0.78 µs. Gain/offset values were 804 (channel 1), 891 (channel S1), 890 (channel D), and 920 (channel 2). Z-step was 0.6–1.0 µm with Nyquist sampling. Channel acquisition was sequential.

Negative controls included the omission of the primary antibody and/or replacement of the primary antibody by purified IgG for the species that was used to raise the primary. The fluorescence signal was determined for experimental localizations with gains of individual channels separately set, yielding no immunofluorescence background for negative controls (2). The exception was the setting for FKBP8 (405), where a clear signal over the background allowed background determination and consequently structure. Quantitative evaluation of the Manders overlap coefficient for co-localization was supported by the Volocity image analysis software, Version 7.0.0.

Primary antibodies included NCL-Hamlet mouse monoclonal for dysferlin targeting synthetic peptide aa 1999–2016 of human dysferlin and crossing to rat dysferlin (Leica, Buffalo Grove, IL, USA); “Romeo” dysferlin rabbit mAb antibody (ABclonal Science, Woburn, MA, USA); TAU mouse monoclonal IgG1 55 clone TAU-5 targeting rat TAU (BD Biosciences Pharmingen, Franklin Lakes, NJ, USA); mouse monoclonal antibody targeting human annexin A2 339-aa fusion protein crossing to rat annexin A2 (66035-1-Ig, Clone No. 1C1E12, Proteintech, Rosemont, IL, USA); annexin A2 affinity-purified polyclonal goat antibody targeting human annexin A2 carboxy terminus crossing to rat (SC-1924, Santa Cruz Biotechnology, Dallas, TX, USA); FKBP8/38 rabbit polyclonal antibody against mouse FKBP8/38 (ab24450, ABCAM, Cambridge, MA, USA); FKBP8 rabbit polyclonal antibody targeting first 355 aa human FKBP8 crossing to mouse (11173-1-AP, Proteintech); FKBP8 goat polyclonal targeting mouse FKBP8 1-326 crossing to rat (PA5-47513; Invitrogen, Carlsbad, CA, USA). Ryanodine receptor (pan) antibody, nominally RYR1, 34C (MA3-925) (Invitrogen, mouse monoclonal); BCL2 mouse monoclonal (60178-1-lg, protein A purified, Proteintech, Rosemont, IL, USA); PSEN1 rabbit polyclonal (ABclonal cat. no. A2187); PSEN1 mouse purified recombinant monoclonal ZooMAb targeting human PS1-Loop crossing to rat (ZMS1110, Sigma-Aldrich, St. Louis, MO, USA); BACE1 rabbit mAb affinity-purified (AA 402-501 human BACE1 crossing to rat (ABclonal); RYR3 rabbit polyclonal affinity-purified Prestige (HPA062004, an ATLAS antibody, Sigma, St. Louis, MO, USA); RYR2 anti-phospho RYR2 rabbit polyclonal (Sigma-Aldrich); RYR1 ARR-001 rabbit polyclonal (Alomone); PSEN1 rabbit polyclonal (ABclonal cat. no. A2187); and APP/β-amyloid mouse MAb against human β-amyloid 1–16 (Biolegend 6E10, 803014).

The primary antibodies were coupled to Molecular Probes/Invitrogen secondary antibodies donkey anti-mouse IgG (H + L) highly cross-absorbed secondary antibody Alexa Fluor 568; donkey anti-rabbit IgG (H + L) highly cross-absorbed secondary antibody Alexa Fluor 488; and donkey anti-goat IgG (H + L) highly cross-adsorbed secondary antibody Alexa Fluor™ Plus 405 (A48259).

### 4.4. Western Blot Analysis

Control (C57BL/6J) and dysferlinopathy (BlaJ–B6.A-Dysf prmd) male mice (7–9 months) served as a source of gastrocnemius muscle. Gastrocnemius muscle was dissected and the myofibers isolated and lysed in HBS buffer containing 0.1% Tween 20 and a protease inhibitor mixture (Promega, Madison, WI, USA) by brief sonication on ice. The lysate was centrifuged at 20,828 g at 4 °C for 10 min. The cleared lysate supernatant was electrophoresed on a 4–12% NuPAGE gel in paired format with control and mutant in adjacent lanes. GADPH was additionally utilized for a loading control. Protein levels in the cleared supernatant were determined with a Qubit fluorometer and equal amounts of protein for control and dysferlinopathy samples loaded on the gels. The gels were transferred to a PVDF membrane, followed by overnight blocking with 5% nonfat milk at 4 °C. Blots were incubated with protein-specific monoclonal and polyclonal characterized primary antibodies overnight at 4 °C. After three 10 min washings with phosphate-buffered saline containing 0.1% Tween 20, the blots were incubated with HRP-conjugated donkey secondary antibodies (1:10,000) overnight at 4 °C and the proteins detected using Western Lightning chemiluminescence (Pierce™ ECL Western Blotting Substrate/ThermoFisher Scientific, Waltham, MA, USA). ImageJ 1.54J was utilized to quantitate densitometric images with GraphPad statistical analysis (both unpaired and paired variate analysis applied), normalized for the total protein in cleared lysate supernatant.

## 5. Conclusions

### Dysferlin Interaction Pathways Underlying Ryanodine Receptor Regulation of Calcium

Dysferlin disposition in the brain, nerves, and muscle in AD appears to be complex in its mechanism. In addition to reports suggesting dysferlin accumulation in nerves in AD [36] are reports of reductions in dysferlin in N2A mouse neuroblastoma cells [54], raising the possibility of cellular redistribution. In s-IBM, a common aging-associated degenerative myopathy [62], dysferlin was found to be reduced in muscle fiber sarcolemma and present instead in intra-myofiber dysferlin-immunoreactive “plaque-like” cytoplasmic aggregates, with no significant difference in the amount of total dysferlin, suggesting a change in cell distribution. The plaque-like dysferlin co-aggregated with Aβ42, and dysferlin was found to be a “co-binding” partner of AβPP by co-immunoprecipitation/immunoblotting, indicating the presence of both proteins in a protein complex, although not necessarily by direct binding. AβPP-overexpressing cultured human muscle fibers yielded the same result [62], which speaks to the issue of dysferlin disposition in muscle in AD. The lack of dysferlin on the sarcoplasmic membrane was cited as possibly contributing to s-IBM abnormal membrane repair, with abnormal dysferlin trafficking and membrane repair [62]. Interestingly, AβPP does directly interact with the channel protein, CNGA3, according to yeast two-hybrid analysis, which has been sourced in the present investigation to mammalian cochlear hair cell stereocilia (Supplementary Figure S1A,B) [27]. The underlying PPI pathways indicate complexities that need to be revealed, particularly as they relate to the modulation of ryanodine receptor function (Figure 8A). Models of dysferlinopathy where the disposition of dysferlin can be assured suggest a link between dysferlin and ryanodine receptors via FKBP8, which in turn is coupled to BCL2, which is highly upregulated in dysferlinopathy and further coupled to a reduction in ryanodine receptor 1 (Figure 8E,F) [63], ameliorative in AD [13], with FKBP8 therefore being potentially therapeutic.

Neurodegenerative diseases are known to be associated with sensory system defects. The current study documents the presence of cochlear proteins that, when mutated, may be involved in Alzheimer’s disease (AD) with hearing deficits. Hearing loss is an early predictor of dementia in Alzheimer’s disease [64]. Auditory molecular links, such as ryanodine receptor targeting [14], TAU phosphorylation [65], amyloid-beta expression [11], and DNA damage [13], are all factors related to Alzheimer auditory deficits.

Mutations of APP and BACE1 can yield AD amyloid-β plaques, which have been shown to accumulate dysferlin [36]. In neurodegeneration, the levels of ryanodine receptors are increased, and BCL2 and FKBP8 are decreased, reflecting dysregulation by RYRs of calcium-dependent calcium release [56,57,58]. Ryanodine receptors have a key role in amyloid-beta production [55], reflected in their elevation in AD. Three main mammalian RYR receptor subtypes exist, RYR1, RYR2, and RYR3, typical of the skeletal muscle, heart, and brain, respectively [66]. RYR3 is particularly associated with cognitive deficits in the brain [59]. In the cochlea, RYR3 is found in synaptic terminals beneath the inner and outer hair cells [53]. RYR2 is located in inner hair cells and supporting cells [53]. RYR1 is localized to cochlear interdental cells [10,53], consistent with possible mechanisms involving RYR dysfunction in the inner ear. Anti-apoptotic BCL2 [14] binds to and inhibits ryanodine receptors [7,8] and is known to form molecular complexes with FKBP8 and presenilins [25]. Accordingly, FKBP8 may inhibit apoptosis by anchoring BCL2 to mitochondria [6]. The reciprocal relation between the levels of ryanodine receptors and the levels of BCL2-FKBP8 suggests a “see-saw” relation, whereby an increase in BCL2-FKBP8 may be associated with a decrease in ryanodine receptors and the amelioration of AD.

Thus, the elevation of ryanodine receptors, reflecting Ca^2+^ dysregulation typical of AD, may be countered by the interventional elevation of anti-apoptotic FKBP8, a binding partner of BCL2, also elevated and positioned to inhibit RYRs. Dysferlinopathy, reflected in a perturbation of the dysferlin PPI pathway, is accompanied by the elevation of BCL2 and a reduction in RYR, an indicator of relative propensities predicting the amelioration of AD. AD/incipient AD is recognized to be associated with the “downregulation of RYR stabilizers” such as FKBP1a (FKBP12). FKBP8, as part of the FKBP complex including FKBP1a, may participate directly in the FKBP modulation of RYR2 in AD [67], in addition to acting through its association with BCL2.

## Data Availability

Essentially all data associated with this paper can be found within the paper itself and the Supplementary Materials.

## Supplementary Materials

The following supporting information can be downloaded at: https://www.mdpi.com/article/10.3390/ijms26199559/s1.
