# Supplementary material for: Dysferlin Protein–Protein Interaction Pathways in the Organ of Corti and Spiral Ganglion Intersect with Alzheimer’s Protein Pathways
Source: Int J Mol Sci. 2025 Sep 30;26(19):9559. doi: 10.3390/ijms26199559 (PMC12524998; doi:10.3390/ijms26199559)
Supplement: Supplementary file 1 [file ijms-26-09559-s001.zip › ijms-3797028-supplementary.docx]

_________________________________________________________________________________________________________________

**
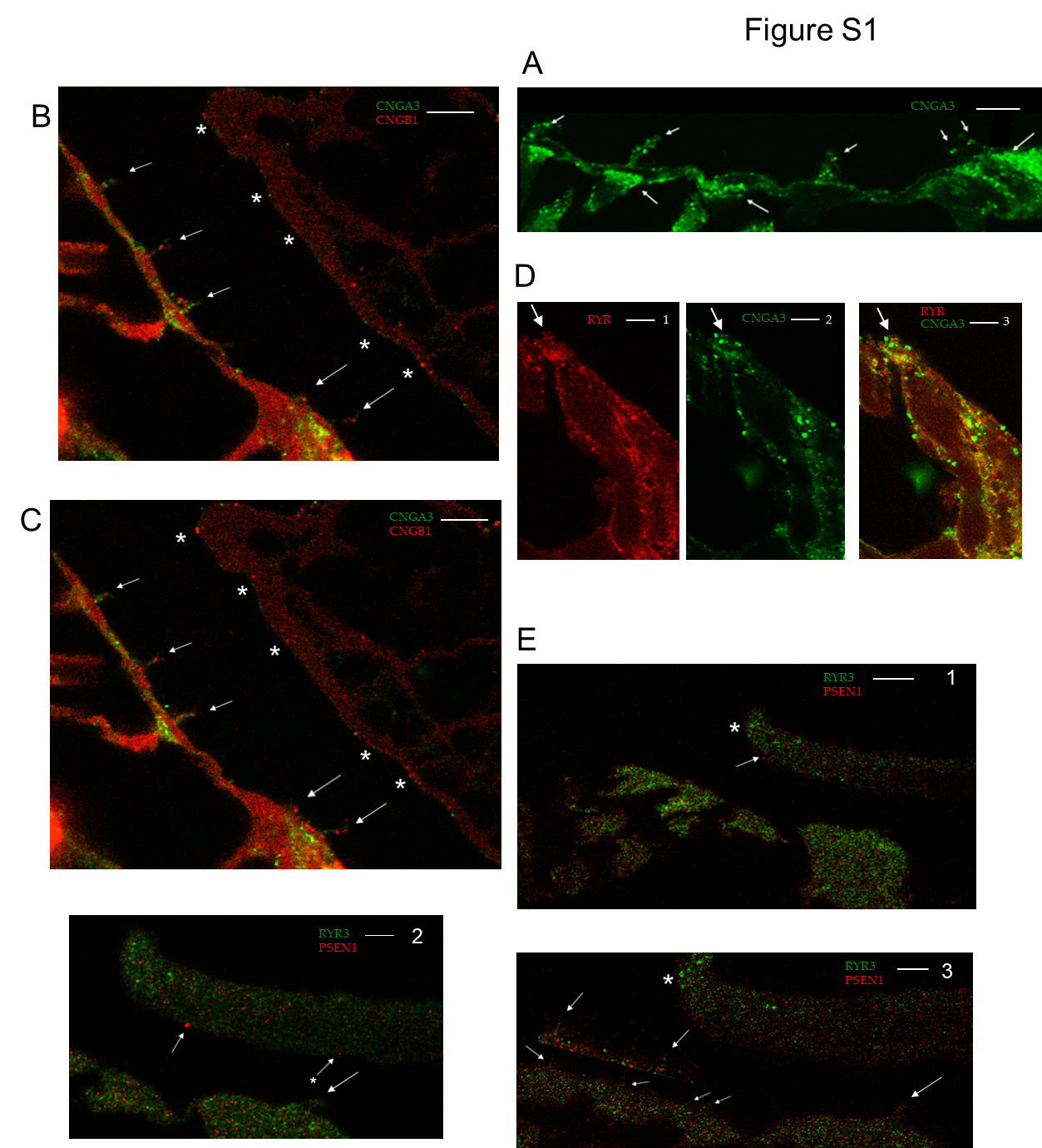
 Supplementary Materials**

**Figure S1**. (**A**) CNGA3 in hair cell stereocilia (short arrows) and in the subcuticular plate of IHC and OHC (long arrows); scale bar = 5 µm. (**B**) Apical turn; Z-stack optical section 9. CNGA3 (green) and CNGB1 (red) immunolocalized to stereocilia of IHC (arrow) and OHC (short arrows) and tectorial membrane (*); Scale bar = 10 µm. (**C**) Z-stack optical section 8; CNGA3 (green) and CNGB1 (red) immunolocalized to stereocilia of IHC (arrows) and OHC (short arrows) and tectorial membrane (*); scale bar = 10 µm. (**D1**) RYR receptor (pan antibody, red). (**D2**) CNGA3 (green) in IHC including IHC stereocilia. (**D3**) RYR and CNGA3. Scale bars for 1-3 = 5 µm. (**E1**) Slide 26 optical section 9: PSEN1 (red, Sigma) OHC stereocilia insert on the TM (arrow); RYR3 in tip of TM (asterisk); scale bar = 5 µm. (**E2**) Optical section 8: PSEN1 (red, Sigma) OHC stereocilia insert on the TM (small arrow); PSEN1 IHC insert on the TM (small arrow + asterisk); IHC stereocilia (large arrow); scale bar = 2.5 µm. (**E3**) Optical section 4: PSEN1 (red, Sigma) and RyR3 (green). PSEN1 (red, arrows) is stacked in OHC stereocilia alternating with RyR3 (green). IHC stereocilia (long arrow); RYR3 in tip of the TM (asterisk). Scale bar = 2.5 µm. Inset (left): magnification of OHC stereocilia.
